# Utility of a Caprini-combined prediction model in patients with gynecological venous thromboembolism in China

**DOI:** 10.3389/fmed.2025.1618023

**Published:** 2025-10-31

**Authors:** Lijuan Ma, Lei Mao, Peipei Jia, Lin Wang, Lili Han, Xiumin Zhang, Ming Hou, Haiyan Ren, Chunyan Yan, Qingfeng Tang, Tao Han, Kereman Yakufu

**Affiliations:** ^1^Department of Gynecology, People’s Hospital of Xinjiang Uygur Autonomous Region, Urumqi, China; ^2^Xinjiang Cervical Cancer Prevention and Treatment Clinical Medical Research Center, Urumqi, China; ^3^Department of Medical, People’s Hospital of Xinjiang Uygur Autonomous Region, Urumqi, China; ^4^Department of Nursing, People’s Hospital of Xinjiang Uygur Autonomous Region, Urumqi, China; ^5^Department of Endocrinology, People’s Hospital of Xinjiang Uygur Autonomous Region, Urumqi, China; ^6^Department of Dermatology, People’s Hospital of Xinjiang Uygur Autonomous Region, Urumqi, China; ^7^Xinjiang Emergency Center, People’s Hospital of Xinjiang Uygur Autonomous Region, Urumqi, China

**Keywords:** venous thromboembolism, Caprini scale, prediction model, gynecological patients, nomogram

## Abstract

**Objective:**

Explored the risk factors for venous thromboembolism in gynecological inpatients in western China, and developed an improved model to predict the VTE of this patient population.

**Methods:**

The records of 6897 patients hospitalized in the Gynecology Department of Xinjiang Autonomous Region People’s Hospital were retrospectively reviewed, during January 1, 2021 to July 31, 2022 and meet the inclusion criteria are selected. The efficacy of the Caprini-combined prediction model was evaluated, and the Caprini-combined prediction model and independent risk factor-combined prediction model for predicting VTE were assessed using receiver operating characteristic (ROC) curve analysis.

**Results:**

The study cohort was divided into two groups: a VTE group (*n* = 229) and a non-VTE group (*n* = 6,668). Univariate analysis was performed on all patients, followed by collinearity diagnostics for variables that showed statistically significant differences. Variables with a variance inflation factor (VIF) <2 were included in the multivariate logistic regression analysis. The results identified the following independent risk factors for VTE: length of hospital stay, age ≥70 years, Caprini score ≥3, respiratory or heart failure, body mass index (BMI) ≥30 kg/m^2^, high platelet count, low serum albumin level, and elevated D-dimer level. Among these, the Caprini score demonstrated the strongest association with VTE (OR = 9.939). The areas under the ROC curve for the Caprini scale, the combined Caprini prediction model (incorporating Caprini score, serum albumin, and D-dimer levels), and the combined model of all independent risk factors were 0.671, 0.973, and 0.979, respectively. We had developed a simple nomogram to predict VTE.

**Conclusion:**

Our study constructed a nomogram for predicting the risk of VTE based on age, BMI, Caprini scale score, respiratory/heart failure, length of hospital stay, PLT, serum albumin, and D-dimer levels. This model has good discrimination and prediction effects, and has certain clinical value.

## 1 Introduction

Venous thromboembolism (VTE) is a thrombotic disease of the venous system that includes deep vein thrombosis (DVT) and pulmonary thromboembolism. Hospitalized patients experience a high incidence of VTE. The average global incidence rate of VTE is approximately 1.17% (1–3). VTE can cause severe survival pressure and economic burden to patients. The National Health Commission has devoted increasing attention to the prevention and control of thrombosis and VTE, and hospitals have incorporated these practices into medical quality management and monitoring systems (4) and are gradually working to strengthen them.

Patients with gynecological diseases often experience lower-limb DVT due to poor deep vein blood flow, with severe cases further progressing to pulmonary embolism (PE), both of which are considered types of VTE (5). Gynecological diseases can cause VTE in patients owing to their unique anatomical location, such as open surgery, malignant tumors, laparoscopic surgery, and hormone therapy (6). Gynecological surgery primarily focuses on the pelvic region. During surgery, many blood vessels can be directly or indirectly injured, and patients are required recover in bed for prolonged periods after such procedures, further increasing the probability of venous thrombosis (7).

Several factors affect venous thrombosis. In 1859, Virchow first proposed that blood stasis, hypercoagulability, and vascular wall damage are the three major factors contributing to thrombosis. Any of these three pathological states is a risk factor for VTE (8). Although several personalized risk models have been developed and clinically evaluated (9, 10), they have not been widely validated (11, 12). Since its revision in 2009, the Caprini risk model has been retrospectively validated in a large Western population, and its effectiveness and feasibility in screening individuals at high risk for VTE has been demonstrated (13). The model includes approximately 40 different risk factors, making the assessment complex and time-consuming. As such, the present study retrospectively analyzed risk factors for VTE in patients with gynecological diseases and designed a warning scoring table based on a risk assessment model that is more consistent with the characteristics of patients undergoing gynecological surgery. Therefore, more targeted assessments and interventions are anticipated.

This study aimed to evaluate the effectiveness of the Caprini scale in predicting VTE in hospitalized patients with gynecological diseases and to design an improved model with a high predictive yield.

## 2 Materials and methods

### 2.1 Study subjects

The present investigation was a retrospective cross-sectional study. Patients who were admitted to the Gynecology Department of Xinjiang Autonomous Region People’s Hospital (Urumqi, Xinjiang, China) between January 1, 2021, and July 31, 2022, and fulfilled the inclusion criteria were selected. The study was approved by the Ethics Committee of the People’s Hospital of the Xinjiang Autonomous Region (No: KY2023041102), and all patients provided consent to participate. During data collection, information was obtained to identify eligible participants. Patients were divided into 2 groups according to whether VTE was diagnosed during hospitalization: VTE and non-VTE. Patients with a Caprini score ≥3 were allocated to the high-risk group, while others were allocated to the low-risk group.

### 2.2 Diagnostic methods for VTE

Deep vein thrombosis (DVT) was diagnosed using deep vein ultrasound and/or deep vein angiography of the lower extremities, and pulmonary thromboembolism was diagnosed using computed tomography pulmonary arteriography. All patients included in this study were diagnosed with symptomatic VTE after comprehensive screening and were confirmed to have clinically suspected VTE (14).

### 2.3 Inclusion and exclusion criteria

Inclusion criteria were as follows: age ≥18 years; hospitalized for gynecological diseases; the length of hospital stay >3 days. Exclusion criteria: Patients who received anticoagulation treatment upon admission, patients who were unable to confirm new VTE during hospitalization, and patients without complete clinical data for evaluation were all excluded.

### 2.4 Classification criteria for gynecological diseases

The classification standards for all gynecological diseases were based on a current internationally recognized classification standard; more specifically, the *International Classification of Diseases, 10^th^ Revision* (i.e., “ICD-10”). Diseases were classified into the following categories: malignancy, benign tumor, hydration, prolapse, uterine polyp, uterine adenomyosis, gynecological inflammation, vaginal bleeding, and other gynecological diseases.

### 2.5 Data acquisition

Eleven risk factors from the Caprini scale, including serum albumin, D-dimer, and platelet (PLT) levels were screened within 1 week before discharge or VTE. Each patient was strictly rated for thrombotic risk according to Caprini scale standards by professionally trained gynecological experts.

### 2.6 Statistical analysis

All statistical analyses were performed using SPSS version 22.0 (IBM Corp., Armonk, NY, USA), R (R Foundation for Statistical Computing, Vienna, Austria)^1^, and EmpowerStats (X&Y Solutions Inc., Boston, MA, USA)^2^. Spreadsheet software (Excel, Microsoft Corp., Redmond, WA, USA) was used to record patient information and establish a database. Measurement data with normal distribution were expressed as x ± s and the *t*-test was used for comparison between the two groups. Measurement data with non-normal distribution were expressed as median (interquartile range [IQR] P_25_ and P_75_), and the non-parametric Mann–Whitney U rank-sum test was used for comparison between the two groups. Categorical data were expressed as frequency (percentage), and comparison between the two groups was performed using the χ^2^ test or Fisher’s exact test, as appropriate. Logistic regression was used to analyze independent risk factors for VTE, and odds ratio (OR) and corresponding 95% confidence interval (CI) were calculated. A nomogram was established based on the results of multivariable analysis (15). The area under the receiver operating characteristic (ROC) curve (AUC) was used to estimate the sensitivity and specificity of the Caprini scale, and an improved model for the occurrence of VTE in patients was used. A model with AUC values >0.7 is considered accurate. Differences among the AUCs for the three models were compared using the Delong method, and differences with *P* < 0.05 were considered to be statistically significant.

## 3 Results

### 3.1 Distribution in VTE and non-VTE groups

From January 1, 2021 to July 31, 2022, a total of 9812 hospitalized patients were selected. Among them, 1966 patients were hospitalized for less than 3 days, 571 patients received anticoagulant treatment upon admission, 312 patients had insufficient personal information and data, and 66 patients were under the age of 18. Finally, 6897 patients who met the inclusion criteria were selected for further analysis, including 229 patients (3.32%) in the VTE group and 6668 patients (96.68%) in the non-VTE group.

In the VTE group, there were 161 cases (70.31%) of DVT, 41 cases (17.90%) of PTE, and 27 cases (11.79%) of DVT combined with PTE. The disease distribution in the two groups is shown in Table 1. Malignancy accounted for the highest percentage (44.98%) in the VTE group. In the non-VTE group, Benign tumor accounted for the highest percentage of 25.69, followed by Malignancy (25.10%).

**TABLE 1:** The disease distribution in the VTE and non-VTE groups (*n* = 6897).

Disease type	VTE group	Non-VTE group	Total
	*n* = 229	*n* = 6668	*n* = 6897
Malignancy, *n* (%)	103 (44.98)	1674 (25.10)	1777
Benign tumor, *n* (%)	52 (22.71)	1713 (25.69)	1765
Hydatoncus, *n* (%)	10 (4.37)	628 (9.42)	638
Prolapse, *n* (%)	26 (11.35)	548 (8.22)	574
Uterine polyp, *n* (%)	0 (0.00)	379 (5.68)	379
Adenomyosis of uterus, *n* (%)	5 (2.18)	345 (5.17)	350
Gynecological inflammation, *n* (%)	0 (0.00)	199 (2.98)	199
Vaginal Bleeding, *n* (%)	5 (2.18)	235 (3.52)	240
Other gynecological diseases, *n* (%)	28 (12.23)	947 (14.20)	975

### 3.2 Analysis of VTE related factors in gynecological inpatients

#### 3.2.1 Analysis of high-risk factors for VTE in gynecological inpatients.

Caprini scores, hospital stay days, active malignancy, previous VTE history, respiratory/heart failure, age, BMI, platelet count, serum albumin and D-D levels were significant differences between the VTE group and the non-VTE group (*p* < 0.05), as shown in Table 2.

**TABLE 2:** Univariate analysis for VTE among the hospitalized gynecology patients (*n* = 6897).

Category, *n* (%)	Option	VTE group (*n* = 229)	Non-VTE group (*n* = 6668)	Total (*n* = 6897)	χ2/*Z* value	*p-*value
Active malignancy, *n* (%)	Yes	99 (43.23%)	1190 (17.85%)	1289 (18.69%)	93.883	<0.001
	No	130 (56.77%)	5478 (82.15%)	5608 (81.31%)		
Caprinin scale	<3	12 (5.24%)	2713 (40.69%)	2725 (39.51%)	116.394	<0.001
	≥3	217 (94.76%)	3955 (59.31%)	4172 (60.49%)		
Previous VTE history, *n* (%)	Yes	70 (30.57%)	1804 (27.05%)	1874 (27.17%)	1.381	0.240
	No	159 (69.43%)	4864 (72.95%)	5023 (72.83%)		
Respiratory/heart failure, *n* (%)	Yes	11 (4.80%)	36 (0.54%)	47 (0.68%)	59.464	<0.001
	No	218 (95.20%)	6632 (99.46%)	6850 (99.32%)		
Acute myocardial/cerebral infarction, *n* (%)	Yes	4 (1.75%)	108 (1.62%)	112 (1.62%)	0.022	0.881
	No	225 (98.25%)	6560 (98.38%)	6785 (98.38%)		
Operation or not	Yes	206 (89.96%)	6066 (90.97%)	6272 (90.94%)	0.277	0.559
	No	23 (10.04%)	602 (9.03%)	625 (9.06%)		
BMI, (kg/m2)	<30	100 (43.67%)	4509 (67.62%)	4609 (66.83%)	57.301	<0.001
	≥30	129 (56.33%)	2159 (32.38%)	2288 (33.17%)		
BMI, (kg/m2)		29.82 ± 4.04	26.09 ± 5.46	26.21 ± 5.46	59.516	<0.001
Hospital stay days		12.78 ± 9.72	8.92 ± 7.67	9.05 ± 8.06	21.541	<0.001
Age, (years)		55.00 (48.00–65.00)	48.00 (42.00–57.00)	48.00 (41.00–56.00)	−9.334	<0.001
Age, (years)	<70	189 (82.53%)	6279 (94.17%)	6468 (93.78%)	51.366	<0.001
	≥70	40 (17.47%)	389 (5.83%)	429 (6.22%)		
PLT ( × 10^∧^9/L)		266.00 (221.00–310.00)	213.00 (171.00–254.00)	–	−12.428	<0.001
Serum albumin (g/L)		40.61 (37.63–43.10)	32.00 (28.00–35.00)	–	−22.659	<0.001
D-D (mg/L)		1.80 (0.44–8.70)	0.58 (0.27–1.59)	–	−9.499	<0.001

Multivariate logistic regression analysis: Prior to conducting multivariate analysis, collinearity diagnosis was performed on variables with statistically significant differences in univariate analysis (Table 3). Variables with variance inflation factor (VIF) <2 were included in the multivariate analysis. Regression analysis results showed that hospitalization days, age ≥70 years, Caprini score ≥3, respiratory or heart failure, BMI ≥30, high platelet count, low serum albumin levels, and high D-dimer levels were independent risk factors for VTE in hospitalized gynecology patients, as shown in Table 3. Caprini score was the most closely related risk factor for the occurrence of VTE (OR = 9.939), as shown in Table 4.

**TABLE 3:** Variable collinearity diagnosis in univariate analysis of VTE in gynecological inpatients.

Category	VIF
Active malignancy, *n* (%)	1.103
Hospital stay days	1.100
Serum albumin (g/L)	1.025
D-D (mg/L)	1.025
Respiratory/heart failure, *n* (%)	1.012
PLT (*10∧9/L)	1.011
Age, (years) (≥70)	1.010
Caprinin scale (≥3)	1.005
BMI (≥30)	1.005

**TABLE 4:** Multivariate binary logistic regression analysis for VTE among the hospitalized gynecology patients.

Risk factors	B	S.E	Wals	df	*p*-value	OR	95% CI
Constant	−37.417	2.084	322.358	1	<0.001	0	
Hospital stay days	0.034	0.011	10.606	1	0.001	1.035	1.014–1.056
PLT (×10∧9/L)	0.019	0.002	72.569	1	<0.001	1.019	1.015–1.023
Serum albumin (g/L)	0.7	0.049	203.319	1	<0.001	2.014	1.829–2.217
D-D (mg/L)	0.241	0.031	62.012	1	<0.001	1.273	1.199–1.352
Active malignancy, *n* (%)	0.366	0.255	2.069	1	0.15	1.442	0.876–2.376
Age, (years) (≥70)	0.972	0.309	9.909	1	0.002	2.643	1.443–4.84
Caprinin scale (≥3)	2.297	0.37	38.437	1	<0.001	9.939	4.809–20.543
Respiratory/heart failure, *n* (%)	1.666	0.691	5.818	1	0.016	5.291	1.367–20.488
BMI (≥30)	0.919	0.214	18.475	1	<0.001	2.506	1.648–3.809

**FIGURE 1:**
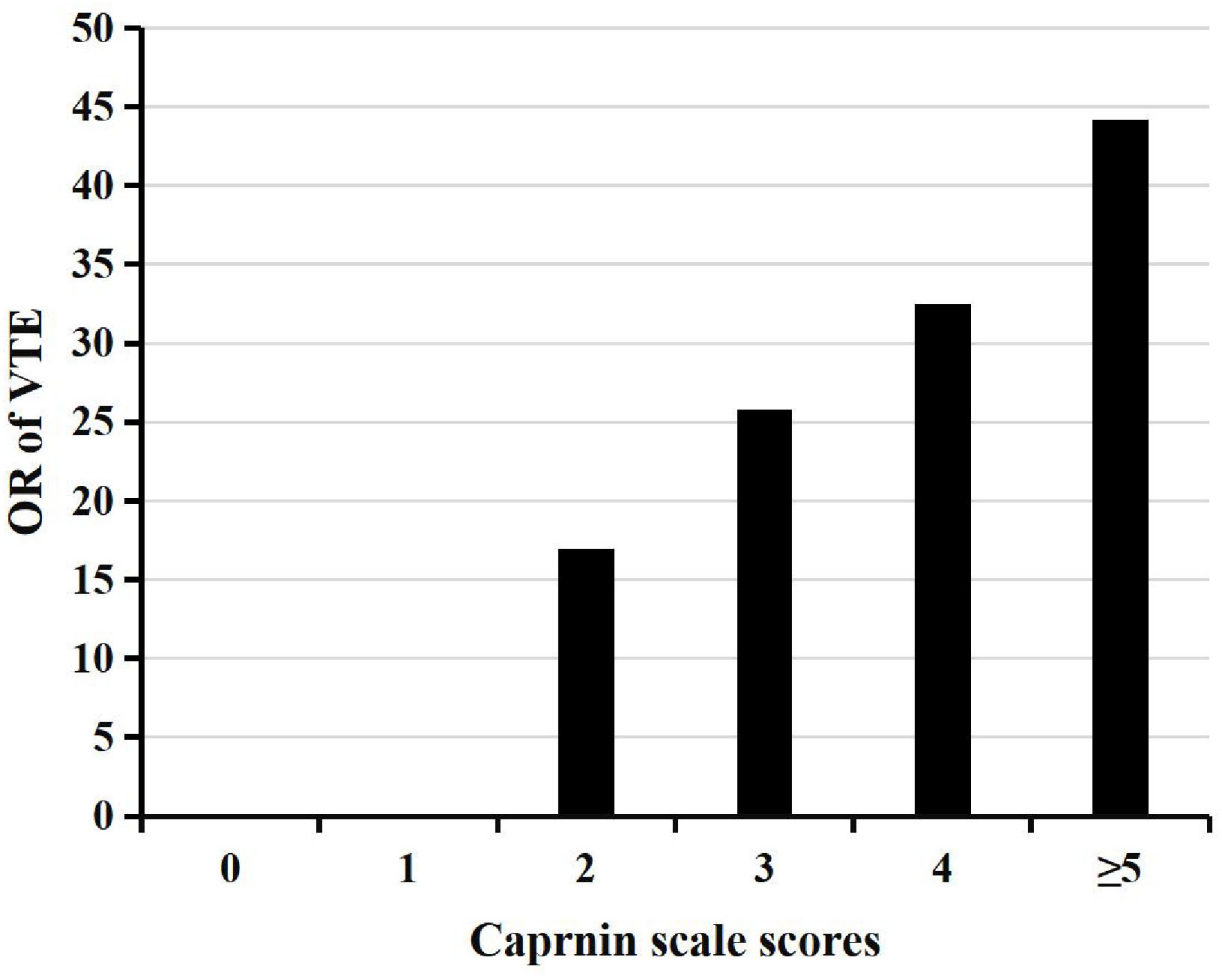
Caprinin scale scores and risk of VTE. VTE, venous thromboembolism; OR, odds ratio.

The higher the Caprinin scale level, the higher the percentage of VTE identification, which was statistically significantly different (χ2 = 154.244, *p* < 0.001). Additionally, with the increase in the Caprinin score, the possibility of VTE occurrence was also higher (shown in Figure 1).

**FIGURE 2:**
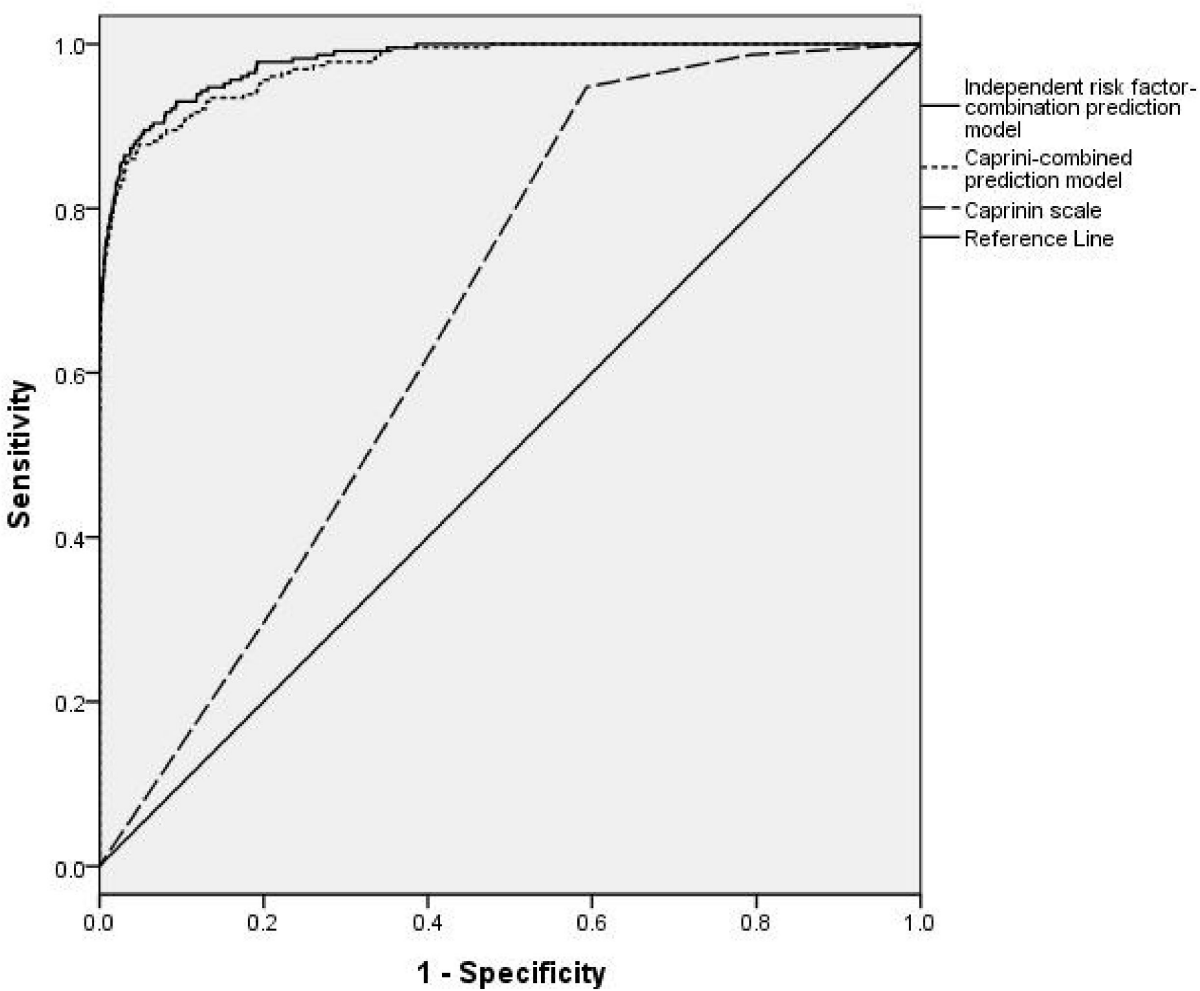
ROC curves of the Caprinin scale, Caprinin combined-prediction model, and independent risk factor-combined prediction model. Caprinin combined prediction model refers to the Caprinin scale combined with D-D and serum albumin. The independent risk factor-combined prediction model refers to the combination of nine risk factors as follows: hospitalization days, age 70 years, Caprini score 3, respiratory or heart failure, BMI 30, high platelet count, low serum albumin levels, and high D-dimer levels.

Caprini scoring prediction model, the AUC was 0.671 (95% confidence interval [CI]: 0.645–0.698), with a Youden’s index value of 0.354 (Sen = 94.76%, Spe = 40.69%), F1 score is 0.399. Caprini-combined prediction model, the AUC was 0.973 (95% CI: 0.964–0.983), with a Youden’s index value of 0.829 (Sen = 87.77%, Spe = 95.17%), F1 score is 0.697. Independent risk factor-combination prediction model, the AUC was 0.979 (95% CI: 0.972–0.987), with a Youden’s index value of 0.841 (Sen = 89.52%, Spe = 94.59%), F1 score is 0.725 (shown in Figure 2 and Table 5).

**TABLE 5:** Results of ROC curves for internal and external validation in males and females.

Model	AUC	95% CI	Youden index	Sensitivity (%)	Specificity (%)	*p*-value	F1 score
Model 1	0.671	0.645–0.698	0.354	94.76%	40.69%	<0.001	0.399
Model 2	0.973	0.964–0.983	0.829	87.77%	95.17%	<0.001	0.697
Model 3	0.979	0.972–0.987	0.841	89.52%	94.59%	<0.001	0.725

Model 1, Caprinin scale; Model 2, Caprini-combined prediction model; Model 3, independent risk factor-combination prediction model.

### 3.3 Usage of nomogram

**FIGURE 3:**
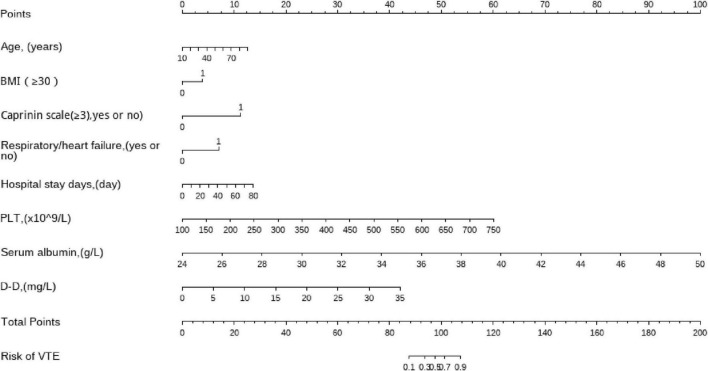
Nomogram to predict the risk of VTE in gynecological inpatients. Draw an upward vertical line from each variable axis to the points bar to get the points of each variable. Based on the sum of each variable points, draw a downward vertical line from Total points axis to calculate Risk of VTE bar.

By taking a gynecological inpatients who is 40 years old as an example, drawing an upward vertical line from the age variable axis to the Point bar gets a point of 5. If her BMI is 31, the point corresponding to the BMI variable is 4. If her Caprinin scale is 4, the point corresponding to the Caprinin scale variable is 11. If her hospital stay is 32 days, she can get 7. If the PLT of this patient is 290 (x10∧9/L), the point corresponding to the PLT variable is 18.5. If the Serum albumin of this patient is 31 g/L, the point corresponding to the Serum albumin variable is 27.5. If the D-D of this patient is 10 mg/L, the point corresponding to the Serum albumin variable is 27.5. The total points equal to 88. So, the risk of VTE is 10% when we draw a straight line from the total point axis to the risk of VTE axis (Figure 3).

## 4 Discussion

Venous thromboembolism (VTE) is characterized by a high incidence rate, poor prognosis, and high mortality. It is a serious complication of hospitalization and has become a global public health problem. Current research has shown that the annual incidence rate of VTE is generally approximately 100/100,000, and the annual incidence rate of VTE in western countries is 108/100,000, with 900,000 VTE cases occurring annually (16). Although the incidence rate of VTE among Asians is lower than that in Western countries, it is on the rise annually (17, 18). A large retrospective study from Hong Kong found that the incidence rate of VTE increased from 28.1/100,000 to 48.3/100,000 from 2004 to 2016, an increase of approximately 1.71 times (19). This study found that the overall incidence rate of VTE among inpatients with gynecological disease was 3.32%, which was significantly higher than reported in other studies (20). For economic reasons, this study only conducted comprehensive screening for symptomatic VTE and may have missed some asymptomatic patients with VTE.

In this study, patients with gynecological malignant tumors experienced the highest proportion of VTE (44.98% [103/229]), which is consistent with other research results (21). Owing to the unique anatomical structure(s) in patients with gynecological disease and the impact of abdominal pelvic surgery, chemotherapy, pelvic radiotherapy, hormone therapy, and other factors, the incidence rate of thrombosis is relatively high at all stages. Relevant survey results have shown that the incidence rate of thrombosis in patients with gynecological malignant tumors is as high as 3–25% (22, 23). Therefore, it is necessary to take reasonable preventive measures to effectively reduce the incidence and mortality rates of VTE, especially in those with gynecological malignant tumors (24). It is clear that standardizing the prevention and management of thrombosis in gynecological patients is crucial.

This study found that the longer the length of hospital stay, the higher the incidence of gynecological-related VTE. The longer the length of hospital stay, the more reduced patient activity, increased bedtime, and increased risk for VTE due to iatrogenic trauma, such as surgery. Patients with malignant tumors in the active stage (25, 26), a history of VTE (27), basic diseases, such as respiratory/heart failure (28, 29), elderly patients over 70 years old (30), obesity (31), and other gynecological conditions are more likely to develop VTE. These results have been confirmed in previous studies.

The occurrence of VTE is a result of an imbalance in the coagulation and anticoagulation systems in the body; moreover, there is also a connection between inflammation and immune system imbalance (32). Based on previous studies, we included some blood monitoring indicators that are prone to activity and can reflect patient physical condition. This study compared PLT count, serum albumin, and D-dimer levels and found that gynecological patients who experienced VTE exhibited higher PLT count, serum albumin, and D-dimer levels than the non-VTE group. D-dimer is the most commonly used biomarker of coagulation and fibrinolytic activation (33, 34). D-dimer testing is recommended in patients with suspected DVT or PE. If the results are normal, acute DVT or PE can be ruled out. The positive and negative predictive values of D-dimer for diagnosing DVT have been reported to be 31.0 and 98.6%, respectively (35). Its negative predictive value is clinically significant and can be used as an exclusion diagnostic standard for DVT and PE. A prospective cohort study involving 15,792 adults found that low serum albumin level is a moderate marker of increased VTE risk (36). In the present study, low serum albumin levels were independently associated with the occurrence of VTE. PLT aggregation is an important factor in thrombosis and hemostasis. Multiple studies have shown that an elevated PLT count is associated with the occurrence of VTE in patients with tumors, and that PLT count may be a predictive factor for venous thrombosis in patients with tumors.

In a study by Khorana et al., PLT count was associated with the risk for VTE after chemotherapy (37). A study involving patients with lung cancer found that the occurrence of VTE was associated with an increase in PLT count (38). In recent years, multiple studies have confirmed that PLT-to-lymphocyte ratio is an independent predictor of VTE in patients with malignant tumors (39, 40).

Presently, there are many biomarkers reported in the literature, such as D-dimer, tissue factors, and gene polymorphisms, which may not be able to accurately predict VTE owing to various factors, such as detection methods and subject ethnicity. This requires the standardization of laboratory testing methods in the search for biomarkers for thrombosis in different populations, and then combine multiple biomarkers to establish a prediction model for VTE. Improving the ability to predict VTE is an important aspect of further exploration.

The most commonly used venous thrombosis risk assessment scales currently include the Caprini and Wells score scales, with the Caprini score being more comprehensive and covering more gynecological surgery-related items. Therefore, the Caprini score is the cornerstone of the most widely used VTE prevention and treatment guidelines for gynecological surgery in the United States (41–43). In our study, the AUC for predicting gynecological VTE using the Caprini alone was 0.671, which is close to the value reported by Tan et al. (44).

In the early stages of VTE occurrence, it is necessary to construct a predictive model for VTE occurrence, which is used to identify high-risk individuals and identify high-risk populations as soon as possible, take personalized health interventions in a timely manner, control risk factors, and prevent VTE occurrence in patients as soon as possible. At present, various VTE risk assessment models have been established both domestically and internationally, including the Caprini Thrombosis Assessment Model (45), the Autar Thrombosis Risk Assessment Scale (46), the Padua Prediction Scoring Scale (47), and the Khorana Risk Scoring Scale (48). Different types of risk assessment forms have different applicability, and currently, the Caprini Risk Assessment Model is the most widely used VTE risk assessment model in China. This study focuses on women in western China and constructs a VTE risk prediction model suitable for this region based on the clustering characteristics of risk factors for VTE patients in this population. Therefore, we included serum albumin and D-dimer levels based on the Caprini score and analyzed the predictive performance of a Caprini joint prediction model. Our results showed that the AUC for the Caprini-combined prediction model was higher than that of the Caprini score (*p* < 0.001), with sensitivity decreasing from 94.76 to 87.77% and specificity increasing from 40.69 to 95.17%. These results indicate that the Caprini combined prediction model outperformed the Caprini score in predicting VTE in gynecological inpatients.

We also present the VTE probability of the statistical prediction model in a graphical manner by drawing a column chart (Figure 3), which can intuitively and vividly evaluate the risk of VTE in women’s patients, providing clues for VTE prevention and precision medical treatment. It is a clinical tool that is easy for patients to understand. There are still areas for improvement in this study. Firstly, the included indicators cannot fully cover all risk factors of VTE, resulting in limited predictive ability of the model. Secondly, the study sample is the gynecological patients in the hospital, and no female patients in other departments were collected, thus the incidence rate of VTE was underestimated; This study did not use an external population for validation, and the extrapolation ability of the VTE risk prediction model was evaluated to be poor. Further research can be supplemented by the above questions to improve the predictive ability of the VTE risk prediction model.

## 5 Conclusion

Our study constructed a nomogram for predicting the risk of VTE based on age, BMI, Caprini scale score, respiratory/heart failure, length of hospital stay, PLT, serum albumin, and D-dimer levels. This model has good discrimination and prediction effects, and has certain clinical value.

## Data Availability

The raw data supporting the conclusions of this article will be made available by the authors, without undue reservation.

## Footnotes

1
http://www.r-project.org/

2
http://www.empowerstats.com
